# An Efficient Flow-Based Anomaly Detection System for Enhanced Security in IoT Networks

**DOI:** 10.3390/s24227408

**Published:** 2024-11-20

**Authors:** Ibrahim Mutambik

**Affiliations:** Department of Information Science, College of Humanities and Social Sciences, King Saud University, Riyadh P.O. Box 11451, Saudi Arabia; imutambik@ksu.edu.sa

**Keywords:** IoT security, behavioral-based intrusion detection, flow-based analysis, anomaly detection, network traffic monitoring

## Abstract

The growing integration of Internet of Things (IoT) devices into various sectors like healthcare, transportation, and agriculture has dramatically increased their presence in everyday life. However, this rapid expansion has exposed new vulnerabilities within computer networks, creating security challenges. These IoT devices, often limited by their hardware constraints, lack advanced security features, making them easy targets for attackers and compromising overall network integrity. To counteract these security issues, Behavioral-based Intrusion Detection Systems (IDS) have been proposed as a potential solution for safeguarding IoT networks. While Behavioral-based IDS have demonstrated their ability to detect threats effectively, they encounter practical challenges due to their reliance on pre-labeled data and the heavy computational power they require, limiting their practical deployment. This research introduces the IoT-FIDS (Flow-based Intrusion Detection System for IoT), a lightweight and efficient anomaly detection framework tailored for IoT environments. Instead of employing traditional machine learning techniques, the IoT-FIDS focuses on identifying unusual behaviors by examining flow-based representations that capture standard device communication patterns, services used, and packet header details. By analyzing only benign traffic, this network-based IDS offers a streamlined and practical approach to securing IoT networks. Our experimental results reveal that the IoT-FIDS can accurately detect most abnormal traffic patterns with minimal false positives, making it a feasible security solution for real-world IoT implementations.

## 1. Introduction

The rapid expansion of the Internet of Things (IoT) is evident in the increasing number of devices, users, and applications across various industries and use cases [1,2]. The IoT has significantly integrated into daily life, from healthcare with wearable technology and remote patient monitoring to smart city initiatives leveraging IoT-based sensors for traffic and environmental data [3,4]. These examples underscore IoT’s broad influence across sectors [4,5].

However, this deep integration also brings substantial security concerns. IoT devices often lack standardization and advanced security features due to resource limitations, making them susceptible to cyberattacks [6,7,8]. The varied nature of IoT devices, compounded by the absence of a unified technology or protocol framework, increases the risk of unauthorized access or disruption of critical infrastructure [7,9]. The expanding security risks in IoT ecosystems highlight the crucial requirement for advanced security needs to safeguard IoT networks.

Among the diverse solutions, Behavioral-based Intrusion Detection Systems (IDS) have emerged as effective tools for enhancing IoT network defenses [10,11]. Behavioral-based IDS continuously monitor network operations to detect anomalies or unusual activities, identifying potential intrusions or attacks by analyzing behavioral patterns. The system plays a critical role in promptly alerting administrators to any suspicious behavior, helping to mitigate security threats [12,13].

Various types of Behavioral-based IDS have been developed, each employing unique methods for safeguarding IoT devices. For instance, Signature-based IDS (SIDS) rely on predefined patterns of known attacks to detect malicious activity [14,15]. In contrast, Anomaly-based IDS monitor deviations from established norms in network behavior, making them effective for identifying new or unknown threats [16,17]. Additionally, AI-driven IDS use machine learning algorithms to continuously refine detection capabilities [18,19], ensuring robust protection against evolving cyber threats.

Despite these advancements, Behavioral-based IDS still face critical challenges. Many approaches depend on large, labeled datasets containing both normal and malicious traffic, which are not only difficult to compile but also resource-intensive to process [20,21]. Additionally, machine-learning-based IDS often require significant computational power, making them impractical for resource-constrained IoT devices [22,23].

To address these issues, this paper introduces the IoT-FIDS (Flow-based Intrusion Detection System for IoT), a lightweight and efficient anomaly detection framework tailored for IoT environments. Rather than relying on traditional machine learning techniques, IoT-FIDS uses flow-based representations to analyze network traffic patterns, focusing on normal traffic to detect anomalies. This method reduces computational overhead and simplifies the detection process, making it suitable for real-world IoT implementations.

Our research demonstrates that the IoT-FIDS efficiently detects abnormal behaviors by examining network traffic without relying on pre-labeled attack data. This novel approach profiles device behavior using flow-based analysis of network packets, providing a streamlined solution for intrusion detection in resource-limited IoT devices. The IoT-FIDS effectively identifies malicious traffic patterns, ensuring robust network security while maintaining minimal false positives. This study aims to address the following pivotal research questions:

RQ1: How effective is the IoT-FIDS in accurately and precisely detecting anomalies and intrusions in IoT environments?

RQ2: Is the IoT-FIDS lightweight enough to serve as a practical and feasible security solution for real-world IoT deployments?

This paper is organized to guide the reader through our research effectively. In Section 2, we introduce the main topic, discuss its importance, and provide the necessary background information. Section 3 explains the methods used to generate flow-based representations and to detect malicious packets. Section 4 covers the data-preprocessing steps, detailing how we processed the datasets, selected relevant packet headers, and optimized flow-based representations for the analysis. Section 5 offers an inclusive analysis of the experiments we conducted and the results we achieved. Lastly, Section 6 addresses the limitations of our study, suggest future research directions, and concludes with our final remarks.

## 2. Background

To provide a comprehensive understanding of Behavioral-based IDS, this section explores their various types and characteristics. We first examine their deployment environments, then analyze the methodologies they employ, such as Anomaly-based Intrusion Detection Systems (AIDS) and Signature-based Intrusion Detection Systems (SIDS). Lastly, we focus on advanced approaches leveraging machine learning.

### 2.1. Deployment Environments of Behavioral-Based IDS

Behavioral-based IDS are essential in contemporary security frameworks, continuously monitoring network and system activities to detect unauthorized access or malicious behavior [20,24]. These systems analyze network traffic and operations to identify anomalies or threats. They can be deployed in two key environments: Host-based IDS (HIDS) and Network-based IDS (NIDS).

HIDS focus on protecting individual devices by monitoring their specific activities. Each host examines system logs, oversees file integrity, and observes application behaviors to detect signs of unauthorized access [22,25]. Farrukh [26] introduced SENIDS, a Self-Evolving Network-based IDS that uses artificial neural networks to enhance real-time threat detection for IoT devices. On the other hand, NIDS monitor network traffic as it passes through routers, switches, and firewalls, searching for patterns that match identified attack signatures or exhibit irregular behaviors [27,28].

Recent advancements have focused on enhancing IDS accuracy while reducing complexity. Farrukh et al. [26] introduced a NIDS combining machine learning and the Arithmetic Optimization Algorithm (AOA), achieving up to 99% accuracy with reduced feature complexity. Hybrid systems that merge HIDS and NIDS offer more robust security, effectively correlating device-specific and network-level visibility to detect malicious patterns [29,30].

### 2.2. Signature-Based vs. Anomaly-Based Behavioral-Based IDS

Behavioral-based IDS methodologies are typically categorized into Signature-based IDS (SIDS) and Anomaly-based IDS (AIDS). SIDS operate by maintaining a repository of known cyberattack signatures. When incoming traffic matches an existing signature, an alert is triggered [29,31]. Anju and Krishnamurthy [32] introduced CBSigIDS, a blockchain-enabled framework that updates attack signatures in a decentralized IoT environment. AIDS, in contrast, establish a baseline of normal network behavior and flag deviations from this baseline as potential threats [29,33].

Diro et al. [34] presented an AIDS leveraging kernel PCA for feature reduction and kernel ELM for classification, efficiently distinguishing between normal and malicious traffic. Hybrid systems that combine SIDS and AIDS provide stronger defenses by using signature matching for known attacks and anomaly detection for new, unknown threats [32,35]. Otoum and Nayak [36] proposed AS-IDS, a framework for IoT environments that combines both methods. It filters network traffic, preprocesses data, and uses a lightweight neural network and Deep Q-learning to detect both familiar and novel threats.

### 2.3. Machine-Learning-Based Anomaly Detection

Anomaly-based IDS (AIDS) can be classified into statistical-based, knowledge-based, and machine-learning-based systems. Statistical-based IDS model normal behavior through statistical data, flagging deviations as intrusions [37]. Knowledge-based IDS compare network traffic against predefined benign patterns [14,34,36].

Machine-learning-based IDS learn from traffic patterns to detect novel threats. They adaptively identify attacks that were previously unseen, making them highly effective for modern cybersecurity challenges [29,33]. Intrusion detection approaches are generally categorized into three primary types: supervised, unsupervised, and semi-supervised methods [38,39].

Supervised methods depend on labeled data to classify traffic as normal or malicious. DeMedeiros et al. [40] applied optimization techniques to enhance detection efficiency while minimizing training time. Unsupervised methods, which do not involve labeled data, analyze patterns to detect outliers and potential threats [21,31]. Nassif et al. [41] developed an unsupervised anomaly detection model using flow-based analysis, designed to detect DDoS attacks in IoT networks.

Semi-supervised methods combine aspects of both supervised and unsupervised techniques, typically using a small set of labeled data and a bigger set of unlabeled data. Shen et al. [42] and Khan et al. [43] introduced SS-Deep-ID, a semi-supervised deep learning model that integrates multiscale residual temporal convolutional layers and traffic attention mechanisms, improving anomaly detection by prioritizing critical data points.

### 2.4. Comparative Analysis of Flow-Based IDS Methods

The primary IDS methodologies—SIDS, AIDS, and machine-learning-based IDS—each offer distinct advantages and limitations. SIDS provide high accuracy for known attacks but are ineffective against new or evolving threats and require constant signature updates. AIDS, while capable of detecting unknown attacks, often suffer from high false-positive rates and increased computational demands, making them challenging for real-time deployment. Machine-learning-based IDS adapt to evolving threats and can provide high detection accuracy, but they come with significant resource consumption, require extensive training data, and often involve retraining as new threats emerge.

Our proposed IoT-FIDS addresses many of these limitations by employing a lightweight, flow-based approach that is specifically tailored for resource-constrained IoT environments. Unlike the methods in [1,44], which rely on complex machine learning models or optimization algorithms (such as neural networks optimized with GSA in [44]), the IoT-FIDS does not require computationally expensive training or optimization phases. The IoT-FIDS reduces the need for constant updates (as required by SIDS), lowers false-positive rates (compared to AIDS), and avoids the computational burden typically associated with machine learning models.

- In contrast to [44], which uses a neural network optimized with GSA for flow-based anomaly detection, the IoT-FIDS uses simpler flow-based profiling without the need for iterative optimization. This makes the IoT-FIDS more suitable for real-time intrusion detection in IoT environments where computational resources are limited.
- Compared to [1], which utilizes machine learning for flow-based anomaly detection in SDN, the IoT-FIDS is better suited for IoT environments because it does not require the extensive training and feature selection processes that machine learning models need.

Table 1 provides a detailed comparison of these IDS methodologies, highlighting their advantages, disadvantages, and how the IoT-FIDS overcomes many of their limitations, particularly in terms of computational efficiency and suitability for IoT environments.

The IoT-FIDS provides a more streamlined and efficient solution by leveraging flow-based profiling without requiring machine learning or optimization algorithms, as seen in methods like those in [1,44]. Its suitability for real-time IoT environments, where computational power is limited, makes it a practical choice for intrusion detection in modern networks. By addressing the limitations of SIDS, AIDS, and machine-learning-based methods, the IoT-FIDS ensures low computational overhead and adaptability to evolving threats.

## 3. Methodology

In line with Baz [45] and Zohourian [46], our approach centers on converting each packet into a unique signature that encapsulates its essential features, using the TCP/IP stack with the HTTP protocol, which is widely adopted in IoT networks for reliable communication. This section provides a detailed explanation of how this transformation aids in detecting packets that deviate from normal behavior. We also introduce a metric to measure the distance between these signatures, which is crucial for assessing the level of maliciousness attributed to a packet.

### 3.1. Flow-Based Representation

The flow-based representation strategy is based on three main principles: communication patterns, service types, and header field information. The goal is to eliminate inherent randomness by consolidating numerous similar packets—those performing the same function—into a single representative form. This approach reduces a large dataset of packets into a smaller set of unique flow-based representations, which serve as baseline profiles characterizing typical packet behavior. These profiles successfully capture the key characteristics of normal activity while minimizing difficulty.

#### 3.1.1. Communication Patterns

Due to their resource limitations and restricted communication capabilities, IoT devices generally interact with a limited quantity of endpoints, each designated for a specific function. These devices exhibit a limited range of communication behaviors, making it easier to classify them. The classification of communication patterns is based on several subcriteria. The first factor is identifying the specific endpoints with which the IoT device communicates, using IP and MAC addresses to specify devices or servers. Next, the direction of communication is determined by whether data packets are inbound or outbound. Additionally, the scope of communication is assessed to determine whether the interaction occurs within a local area network (LAN) or extends to external networks (WANs). Finally, the distribution of messages is analyzed to classify whether communication is unicast (to a single recipient), multicast (to multiple recipients), or broadcast (to all devices on the network).

By assessing these factors, a baseline for normal communication patterns is established. Anomalous behavior is identified when a device interacts with a new or unexpected endpoint—whether on a LAN or WAN—or when it communicates with an identified endpoint in an unusual manner. Detecting these anomalies is essential for identifying potential security threats or operational issues within IoT networks.

#### 3.1.2. Service Types

IoT devices rely on specific protocols and port records to manage communication with their endpoints. To distinguish between different service types, three key elements are examined. The first element is the communication protocol in use, such as UDP or TCP. The second element is the assigned port number that facilitates the communication service. Lastly, the specific service being utilized for communication, such as HTTP or DNS, is analyzed. If a device engages with an unfamiliar service or uses an identified service through an unusual endpoint, such activity is highlighted as an anomaly.

#### 3.1.3. Header Field Information

Another important observation is that IoT devices tend to use precise packet header field values consistently through communication. These header values, which vary across network layers, help to classify different packet structures. For Layer 2 protocols, such as ARP, distinct field values are used to identify specific communication traits. Similarly, Layer 3 protocols, including IP, IGMP and ICMP, have distinct field values. At Layer 4, protocols like UDP and TCP exhibit unique header field values, and at Layer 5, protocols such as DNS and HTTP contribute further distinguishing characteristics.

By analyzing the header values across these network layers, the system can effectively classify packets. Any packet that deviates from these established header field patterns is considered irregular and may indicate potential malicious behavior.

### 3.2. Flow-Based Translation

This section outlines the process of converting packets into flow-based representations. Let P  be the collection of all packets, where p∈P  represents a packet. We state p as a sequence of header fields and a load:

(1) $$p=\langle h_{1},h_{2},h_{3},\ldots ,h_{n},d\rangle$$

where $h_{i},d\in {\mathbb{Z}}^{+}$ are positive integers. Let R be the set of flow-based symbols, with r∈R being the symbol of packet p:

(2) $$r=\langle h_{1}^{*},h_{2}^{*},h_{3}^{*},\ldots ,h_{m}^{*}\rangle$$

In this representation, each header $h_{i}$ can be either discarded, retained, modified, or replaced with another value, while the payload d is omitted. The headers chosen for these flow-based representations are elaborated on in Section 4.3. Importantly, m is less than or equal to n, as the goal is to reduce the complexity of and variability in the packets by summarizing them into more concise representations. Ideally, we aim to ensure m is much smaller than n. We define a mapping function f:P→R and for every flow $f\left(p\right)=r$, there exists a corresponding flow-based representation $\forall p\in P,\exists r\in R,f\left(p\right)=r$.

$P_{D}$ is the set of typical packets associated with a specific device D. Each packet in $P_{D}$ is assigned to its equivalent representation, creating the normal profile $P_{D}$, which is the set of standard representations for device D. The total number of representations is expressively fewer than the number of individual packets since we aim to minimize the number of distinct header values. In other words, multiple packets can be mapped to a single representation that captures their key characteristics for that device.

From an execution perspective, the mapping process can be viewed as a sequence of removal, insertion, and transformation steps, converting a packet into its representation. This reduction in features, and thus in representations, is what makes the model lightweight.

For an incoming packet p from device D, the packet is mapped to its representation r, where $r=f\left(p\right)$, the packet is classified as normal. If not, it may be highlighted as anomalous, depending on the representation distance discussed in below.

### 3.3. Deviation in Representation

As outlined earlier, a packet might diverge from typical patterns due to three key factors: communication patterns, services used, and header configurations. For example, irregularities can occur when communicating with an unknown endpoint, using a service not previously encountered, or employing a different header setup. The complexity increases when these issues intersect. For instance, if a device simultaneously communicates with an unknown endpoint, uses a new facility, and adopts a different header construction, the likelihood of anomalies increases.

However, it is crucial to recognize that deviating from any of these factors does not automatically indicate abnormal behavior. In some cases, it could just be an unfamiliar, yet legitimate packet inside the bounds of regular traffic. Consequently, simply determining if a packet fits within the usual representation profile is insufficient for identifying abnormalities. Doing so could result in a significant number of false positives. To tackle these challenges and minimize false positives, we introduce a metric that measures the distance between two packet representations, which quantifies the level of abnormality when a packet does not align with any pre-established usual profiles. This metric is key to distinguishing genuinely irregular packets (true positives) after those that might then be mistakenly flagged as false positives.

We use the Hamming distance to determine the number of differences between two representations. Let us assume that u,v are two flow-based representations belonging to the set u,v∈R. We define the distance between them as follows:

(3) $$d\left(u,v\right)=\sum_{i=1}^{n}\left(u_{i}\neq v_{i}\right)$$

Next, the proximity of a packet to a specified set of representations is defined as the shortest distance from any representation in that set. Let $R_{D}$ represent the set of usual representations for device D, and let p represent a new flow with representation u. We describe the distance as follows:

(4) $$d\left(u,R_{D}\right)=\underset{v\in R_{D}}{min}\left(d\left(u,v\right)\right)$$

We utilize this distance metric to assess whether a packet is abnormal, which helps in minimizing false positives caused by insufficient packet data from a specific device.

### 3.4. Profiling and Monitoring

The model operates in two main stages: Analysis and Monitoring. In the Analysis phase, normal network traffic is associated with corresponding models, which are stored in a device profile database. In the Monitoring phase, received packets are compared to the stored representations, and the distance between the packet and the profile is calculated. If this distance exceeds a predetermined threshold, the packet is flagged as suspicious or anomalous.

To further reduce false positives, we implemented flow-based intrusion detection, leveraging packet representations by analyzing the approach of packets within individual flow. This method is advantageous because it evaluates the collective behavior of packets within the same flow, determining abnormality through a consensus mechanism among the individual packets. From an intrusion detection standpoint, generating an alert for every packet is not ideal, as it can overwhelm the user. The outcomes, discussed in Section 5, demonstrate that flow-based intrusion detection significantly enhances performance.

### 3.5. Legitimate vs. Malicious Network Traffic

Legitimate network traffic refers to authorized and non-harmful interactions of data within a network. This type of traffic includes regular, permitted interactions between devices, wherever data packets are created and sent based on standard protocols and normal communication patterns.

On the other hand, malicious network traffic involves unauthorized and harmful data exchanges within a network. These actions are intended to breach the network’s integrity, confidentiality, or availability, potentially affecting connected devices.

The perspective of network packet capture (pcap) reveals that the main difference between legitimate and malicious pcaps is that attack traffic may consist of both valid and harmful packets. Typically, datasets classify a pcap as either fully legitimate or fully malicious, but in reality, a malicious pcap can include both benign and harmful packets. This leads to ongoing discussions about how to properly distinguish between legitimate and attack pcaps. A central issue is the proper labeling of packets or flows in network traffic to accurately identify their malicious nature.

This research, therefore, emphasizes the exact detection of harmful packets inside network traffic, regardless of whether the entire traffic flow has been categorized as legitimate or malicious. The goal is to thoroughly recognize each irregular and potentially harmful packet within the traffic.

## 4. Data Preprocessing

### 4.1. Dataset Overview

This research employs two well-acknowledged datasets: UNSW-NB15 and BoT-IoT. The UNSW-NB15 dataset includes various types of network traffic such as active, power-related, idle traffic patterns and interactive, all of which are incorporated into our study. Meanwhile, BoT-IoT primarily provides normal network traffic, allowing for the establishment of a baseline for typical device and network behavior. The normal packets from both datasets are converted into flow-based representations and cataloged as profiles for the respective devices.

### 4.2. Data Acquisition and Utilization

The UNSW-NB15 dataset assists primarily in profiling network traffic generated by IoT devices, providing packet captures that facilitate detailed behavioral analysis. This dataset covers multiple types of traffic, such as active, power-related, idle, scenario-specific traffic, interactive. Due to this broad coverage, the UNSW-NB15 dataset provides a crucial foundation for establishing a starting point representation of IoT device behavior. This dataset was gathered from a consistent network environment, and the normal traffic from UNSW-NB15 is used to establish this baseline profile.

In contrast, BoT-IoT is an attack-focused dataset containing packet captures of both benign and malicious traffic generated by IoT devices. It supports a wide range of security analyses, offering different types of attacks, such as reconnaissance, spoofing, web-based threats, and DDoS. In this study, we begin by leveraging the benign traffic from BoT-IoT to strengthen the baseline profile of normal behavior. Subsequently, the dataset’s attack traffic is applied during the monitoring phase to facilitate Behavioral-based IDS analysis.

### 4.3. Packet Header Optimization

A systematic approach was undertaken to carefully select the most suitable packet header values for mapping purposes. Initially, the packets included all header values from the Ethernet, UDP, and IP, as well as TCP layers. Gradually, irrelevant or ineffective headers were eliminated, and the process was broken down into several steps.

#### 4.3.1. Elimination of Random Headers

Certain packet headers exhibit randomness and offer little insight into the behavior or identification of devices. For instance, headers related to length and size fluctuate based on network conditions and the volume of transmitted data, making them appear arbitrary from an application’s perspective. Additionally, sequence numbers, offsets (based on packet segmentation), and checksums used for data truthfulness verification do not reflect precise device patterns.

#### 4.3.2. Removal of Static Headers

To focus on distinguishing features in flow-based analysis, static headers that remain constant due to the simplicity of IoT device behavior or the nature of network protocols were removed. Since this study focused exclusively on UDP and TCP packets, fields like category in the ETH layer and type and protocol in the IP layer were deemed irrelevant. Additionally, headers showing no significant variation across the dataset were found to lack differentiating potential. As a result, headers such as type, ip-hdr-len, diff-ser-field, version and protocol were excluded.

#### 4.3.3. Transformation of Headers with Variable Values

Port numbers are essential for identifying a device’s typical services. However, client-side ephemeral ports are randomly chosen for each TCP/UDP session, leading to excessive variability and poor scalability. Some operating systems also limit ephemeral port selection to specific ranges. To address this, ephemeral ports were grouped into ranges of 5000 (e.g., [0–5000], [5001–10,000], etc.). Likewise, numerous IoT devices display consistent payload lengths, which can act as identifying features. To ensure uniformity, the payload lengths for both UDP and TCP were rounded up to the nearest power of 2.

#### 4.3.4. Exclusion of Unstable Headers

Certain headers, such as the source and destination IP addresses, were found to be unreliable because they can change over time. Therefore, these fields were excluded. However, these fields were used to derive a new feature called “scope”, which determines whether the communication takes place locally inside the network or externally over the internet. This newly created “scope” feature was incorporated into the final flow-based representation.

#### 4.3.5. Retention of Communication-Specific Headers

Packet communication is a crucial aspect of this analysis; so, essential headers were retained to capture information related to endpoints, communication direction, scope, and distribution. To achieve this, we retained the MAC addresses of both the source and destination, in addition to the previously developed “scope” attribute. These MAC addresses help retain details about communication endpoints, direction (source to destination), and the distribution method (whether unicast, multicast, or broadcast), as certain MAC address ranges are reserved for multicast and broadcast communications.

## 5. Evaluation

This section provides a detailed description of our strategies for profiling and monitoring network traffic. Our methodology is divided into two primary phases. First, during the profiling stage, we collected and analyzed all distinctive features of packets from usual network activity. This allowed us to build a starting point profile that characterizes typical network behavior. This baseline is crucial as it serves as a reference point for the next phase. In the subsequent monitoring phase, we examined incoming packets and compared their characteristics to the established baseline profile. Based on the degree of their deviation from this baseline, we classified the packets as either normal or abnormal. The core concept of our approach is to utilize the critical information extracted from normal packets as a standard. This enables us to identify and flag packets that significantly diverge from expected patterns, thereby detecting anomalies within the network traffic.

### 5.1. Profiling

In the profiling stage, each packet from the UNSW-NB15 and BoT-IoT datasets was transformed into a distinct, unique flow-based representation. These representations were archived to build an established benchmark of standard network activity. Table 2 illustrates the total number of packets and their corresponding unique representations. This conversion process reduces the packet space through a more streamlined and effective technique.

The number of unique flow-based representations is influenced by the complexity of the features included and how they are transformed. For instance, retaining detailed features such as precise port numbers or packet measurements increases the quantity of representations, while simplifying or removing specific features reduces it. These representations form the search space used by our model to compute distances between packets, directly impacting its efficiency.

Striking the right balance between the complexity of the flow-based representations and their ability to generalize is essential. Achieving this balance ensures accurate and efficient anomaly detection without overcomplicating the model or sacrificing performance.

### 5.2. Labeling

The Behavioral-based IDS developed in this research relies on a detailed analysis of individual network packets, making accurate labeling of the datasets critical. For new attack .pcap files, approximately 94% of packets transmitted from the attacker to the victim were labeled as an “attack”. This decision was guided by a comprehensive understanding of both the entities involved and the nature of the attack. However, it is important to recognize that not all communications between the attacker and victim are necessarily malicious. In some cases, normal interactions may have occurred before the attack. As a result, the labeling process was approached with meticulous care, ensuring a high level of precision in differentiating between legitimate and malicious traffic.

### 5.3. Monitoring

In the monitoring stage, new network traffic, including attack traffic from the BoT-IoT dataset, was evaluated. Initially, normal traffic profiles were constructed using standard data from the UNSW-NB15 and BoT-IoT datasets during the training phase. When new packets arrived, each packet was mapped to its distinctive flow-based representation and compared to the established baseline profiles to detect anomalies.

The evaluation of these profiles against the attack data from BoT-IoT took into account two important aspects. The first was the protocol focus, as the tests were limited to attacks involving TCP or UDP packets. The second aspect was the detection methods, where the model was evaluated using together packet-based and flow-based intrusion detection techniques.

For an individual type of attack, the evaluation was conducted in four scenarios. These scenarios included profiling with UNSW-NB15 data using packet-based intrusion detection, profiling with UNSW-NB15 data using flow-based intrusion detection, profiling with BoT-IoT data using packet-based intrusion detection, and profiling with BoT-IoT data using flow-based intrusion detection.

To ensure the model’s generalization ability was not compromised, the attack data were capped at a maximum of 12,000 packets. Across the majority of cases, the results remained consistent, confirming the robustness of the model under varying conditions.

### 5.4. Evaluating the Effectiveness of IoT-FIDS

IoT-FIDS functions by establishing a behavioral profile for each IoT device, capturing flow-based representations of their typical network packets. When new traffic is detected, the system compares it against the device’s normal profile. Significant deviations from these established patterns are flagged as abnormal or malicious. The system takes advantage of the inherent simplicity and predictability of IoT devices’ behavior and repetitive traffic patterns aligned with their specific functions. Any substantial deviation from this norm is considered suspicious. For instance, if an IoT camera that usually communicates with cloud services over HTTPS starts sending or receiving SSH packets—a protocol not typical for its operation—the system identifies this as anomalous and labels the SSH traffic as malicious.

In this section, we conduct a comprehensive performance analysis of the IoT-FIDS using standard evaluation metrics such as accuracy, precision, recall, and F1-score. We detail the experimental procedures and present results for various attack scenarios, with web-based attacks, reconnaissance efforts, and dictionary brute-force attempts, as well as both Denial of Service (DoS) and Distributed Denial of Service (DDoS) attacks.

#### 5.4.1. Web Attack Detection Results

This section presents our analysis of web attack detection. For each attack scenario, we processed the associated .pcap file through our model, conducting intrusion detection at both the packet and flow-based levels. The outcomes are summarized in Table 3. When employing the BoT-IoT dataset, we observed a notable enhancement in overall performance. This improvement is attributed to the dataset’s comprehensive inclusion of device representations, which the UNSW-NB15 dataset lacks for many devices found in the BoT-IoT version. Despite the limited device information in UNSW-NB15, the profiling model based on this dataset still achieved commendable attack detection, with a recall rate of at least 91%. However, this came with the drawback of a higher number of false positives compared to that when using the newer dataset.

A key finding is that flow-based intrusion detection outperforms packet-based detection. This superiority arises from aggregating packet classifications within a flow and determining the most frequent classification (mode). This method effectively reclassifies packets that were individually marked as regular but are part of a movement where the majority are abnormal. Consequently, flow-based detection attained a perfect recall rate of 100% for all attacks, regardless of the baseline dataset used.

**Figure 1:**
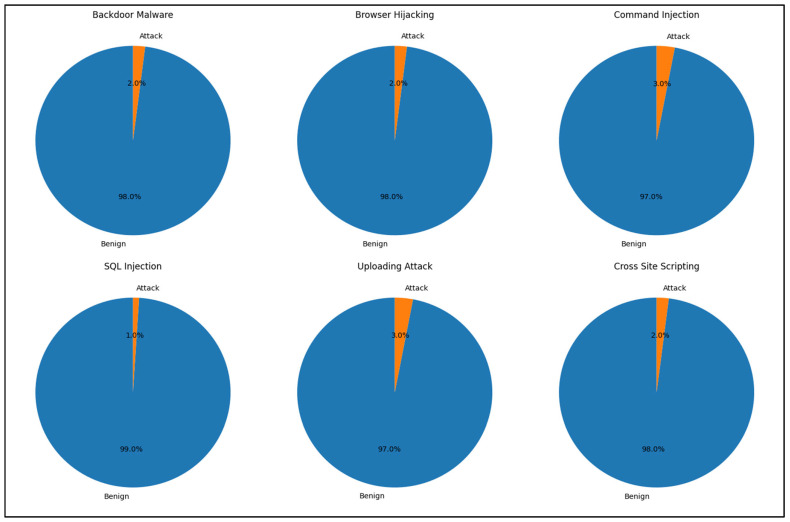
Distribution of benign vs. attack traffic for various web attacks.

Figure 1 illustrates the distribution of benign versus attack flows across all web attack scenarios. The data clearly indicate a significant imbalance, with benign traffic overwhelmingly outnumbering attack traffic. Nonetheless, our model accurately detected all attacks. This outcome underscores that our packet-level attack detection approach remains effective even without balanced datasets.

#### 5.4.2. Reconnaissance Attacks

In the BoT-IoT dataset, each reconnaissance attack type is provided in its own .pcap file, encompassing multiple skimming attempts on various hosts. We assessed our model against all the attacks listed, with the exception of the Ping Sweep attack, which utilizes ARP/ICMP protocols and falls outside the scope of our study.

The results, summarized in Table 4, reveal a pattern similar to that observed with web attacks. Specifically, the BoT-IoT dataset demonstrates superior performance compared to the UNSW-NB15 dataset, and the flow-based method generally outperforms the packet-based method. Despite this, each attack type achieved at least 91% recall and 80% precision. In general, nearly all malicious flows were identified, resulting in only a minimal number of false positives.

#### 5.4.3. Dictionary Brute-Force Attacks

An in-depth examination of the BoT-IoT dataset revealed that the single .pcap file provided for all dictionary attacks actually contains five distinct attacks merged together: three SSH brute-force attacks and two RTSP URL brute-force attacks. This discovery was made possible by sorting packet data based on time deltas using Wireshark. Consequently, we tested each of these five attacks individually using our model, and the detailed outcomes are presented in Table 5. The UNSW-NB15 dataset also exhibited distinct performance, and our evaluations showed that the model performed better overall with the BoT-IoT dataset, particularly in flow-based detection scenarios.

Although the detection rates were exceptionally high, the initial two attacks resulted in a high number of false positives, which significantly reduced precision. A deeper analysis revealed that the false positives were caused by a laptop within the network, which generated packets with inconsistent configurations. This finding underscores that our method is particularly effective for IoT devices, which typically generate straightforward network traffic patterns.

#### 5.4.4. Detection Performance on DoS Attacks

Due to their exceptionally high transmission rates, DoS attacks can be identified using a variety of detection methods. In this study, we evaluated our model on multiple DoS attack types present in the UNSW-NB15 and BoT-IoT datasets. The results, displayed in Table 6, demonstrate that our model achieved high detection performance across the evaluated attacks. This is particularly notable given that both datasets tend to have large amounts of attack traffic relative to normal traffic, creating an inherent imbalance.

To mitigate the effects of this imbalance, we limited our analysis to a sample size of 10,000 packets per attack. Despite this restriction, our method successfully detected nearly all abnormal network flows while maintaining a high accuracy and F1-score for both intrusion detection techniques that analyze individual network packets and those that focus on network flow patterns. Moreover, the false positive rate remained low, showcasing the model’s robustness in distinguishing legitimate traffic from attack traffic, even under challenging conditions.

The analysis revealed that SYN Flood and UDP Flood attacks were detected with near-perfect precision and recall, while more complex attack types like HTTP Flood achieved high detection rates, particularly in flow-based evaluations. These outcomes highlight the usefulness of the model in handling various types of DoS attacks across both datasets.

**Figure 2:**
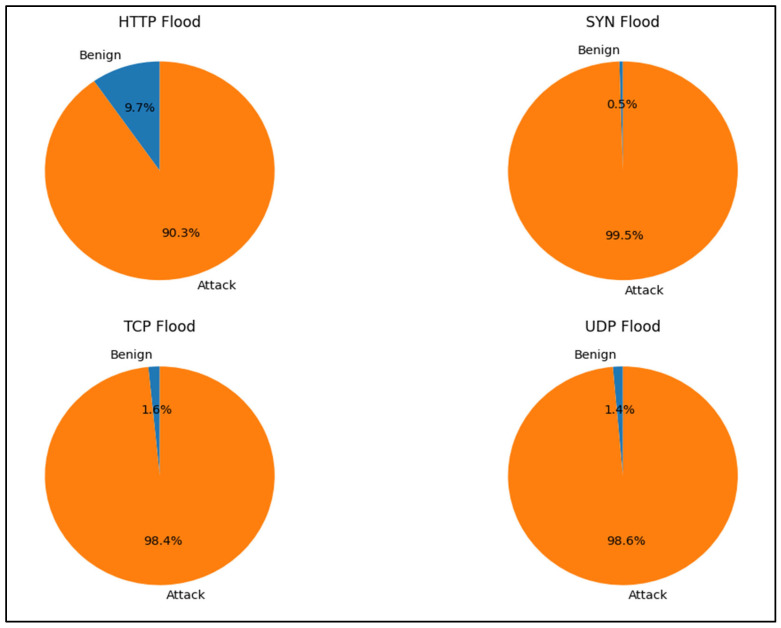
Distribution of benign vs. attack traffic for DoS attacks (UNSW-NB15 and BoT-IoT datasets).

Figure 2 illustrates the distribution of benign versus malicious data for DoS attacks. Given that DoS attacks generate significantly higher volumes of attack traffic compared to normal traffic, we report that our statistical analysis depends on the number of network flows rather than individual packets. It is important to note that the imbalance is even more pronounced at the packet level. Nevertheless, our model consistently achieves high accuracy in detecting attacks, even when the dataset is heavily skewed towards malicious traffic.

#### 5.4.5. Assessment of DDoS Attack Detection

To broaden our experimental evaluation, we incorporated Distributed Denial of Service (DDoS) attacks from the BoT-IoT dataset. We intentionally omitted the identical IP flood attack because it uses the victim’s IP address as both the source and destination, making it easily detectable through basic rule-based methods. Our focus remained on more complex UDP-based and TCP attacks. The outcomes of these experiments, as presented in Table 7, demonstrate a strong performance of our model across various DDoS attack types, with notable metrics such as high accuracy, precision, recall, and F1-scores for attacks like ACK Fragment, SYN Flood, and TCP Flood.

Similar to our observations with DoS attacks, DDoS attacks exhibit high transmission rates that can affect detection performance. We noticed a decrease in detection effectiveness for HTTP Flood and SlowLoris attacks when profiling was based on the BoT-IoT dataset. Further investigation revealed that these attack samples included legitimate communications between the attacker and the victim. This resulted in attack patterns being present in the normal traffic profiles, compromising detection accuracy.

This highlights the critical importance of using clean normal data—completely free of any attack traffic—for accurate profiling. This necessity explains the superior performance achieved when using profiles from the 2022 dataset. Despite these challenges, other instances of HTTP Flood and SlowLoris attacks targeting different endpoints were detected with excellent accuracy.

### 5.5. Evaluating the Operational Efficiency of IoT-FIDS

This section evaluates the practicality of IoT-FIDS for real-world deployment by analyzing network traffic durations and detection times. The assessment was carried out in a network environment with approximately 80 endpoints, encompassing different types of Ethernet addresses such as unicast, multicast, and broadcast. Table 8 provides a breakdown of the number of endpoints, the duration of the exam traffic, and the detection times for each type of attack based on the UNSW-NB15 and BoT-IoT datasets. The outcomes indicate that IoT-FIDS demonstrated consistently low detection times across various attack types, supporting its ability to effectively monitor real-time traffic and promptly detect anomalies. This performance highlights the IoT-FIDS’ suitability for resource-constrained IoT environments, where minimizing detection time is crucial for maintaining network security. When compared with more computationally intensive methods, the IoT-FIDS’ lightweight, flow-based approach reduces overhead, making it more efficient for real-time applications without sacrificing accuracy. These findings further reinforce the IoT-FIDS’ capability to serve as a practical and reliable intrusion detection system in diverse IoT settings.

**Figure 3:**
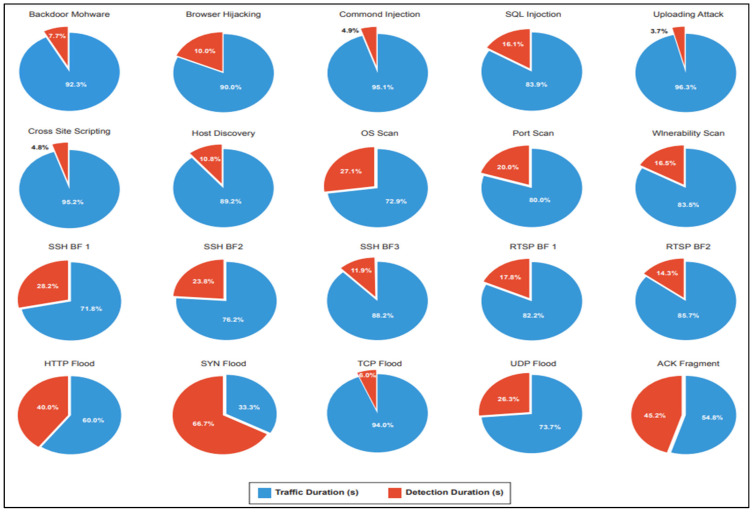
Comparing traffic and detection duration for various attack types.

For enhanced clarity and comparison, Figure 3 illustrates these metrics graphically. The findings clearly demonstrate that, for the majority of attacks, the detection times are significantly shorter than the network traffic durations. This indicates that the IoT-FIDS is sufficiently lightweight for deployment in real-world applications, enabling near real-time Behavioral-based IDS. The execution time encompasses several processes: mapping packets to their respective flow-based representations, computing distances between these representations to identify malicious packets, and ultimately detecting all malicious network flows grounded on packet-level examination.

#### 5.5.1. Analysis of Mapping and Feature Extraction Time Complexities

Let us consider a benign network traffic dataset consisting of n  packets. The total time required for mapping and feature extraction processes can be expressed as follows:

(5) $$T_{mapping}=n\cdot T_{p\to r}=n\cdot T_{p\to v}=T_{featureExtraction}$$

where

- $T_{p\to r}$ represents the time required to convert a packet into its representation.
- $T_{p\to v}$ signifies the time needed to derive characteristics from a packet.

Both processes share the same time complexity because we engineer and select various features from every packet, leading to a linear time complexity of $O\left(n\right)$ for both IoT-FIDS and autoencoder-based methods.

#### 5.5.2. Profiling and Training Time Complexities

During the profiling phase, the system simply stores the flow-based representations of packets. This operation does not depend on the number of packets and thus has a constant time complexity of $O\left(1\right)$. However, training an autoencoder introduces significantly higher computational demands. The total training time can be calculated as follows:

(6) $$T_{training}=n\cdot \left(T_{FeedForward}+T_{ReconstructionError}+T_{BackPropagation}\right)\leq n\cdot \left(O\left(n^{2}\right)+O\left(n\right)+O\left(n^{2}\right)\right)\leq O\left(n^{3}\right)$$

Explanation:

- Feedforward complexity arises from computing the activations in each layer.
- Reconstruction error complexity refers to the error calculation between the input and output.
- Backpropagation complexity involves updating the weights, which adds to the computational burden.

This indicates that the training time for an autoencoder increases cubically with the number of packets, making it computationally intensive for large datasets. In contrast, the IoT-FIDS requires no training, making it more efficient in environments where real-time performance is essential.

#### 5.5.3. Monitoring and Testing Time Complexity Analysis

When analyzing unknown network traffic consisting of n packets, each with mmm extracted features, the monitoring phase of the IoT-FIDS system involves two main steps for each packet: mapping it to a flow-based representation and calculating its distance to stored representations from the profiling phase. The total time required for these operations is as follows:

(7) $$T_{profiling}=n\cdot T_{p\to r}+n\cdot T_{distance}=n\cdot T_{p\to r}+n\cdot k\cdot m=O\left(n\right)+O\left(n\cdot k\cdot m\right)\leq O\left(n^{3}\right)$$

Let k represent the maximum size among all sets of flow-based representations within the device profiles. For every individual packet, we start by mapping it to its specific representation. We then compute the distance using Equation (1) by discovering the smallest Hamming distance between this packet’s flow-based representation and the normal representations associated with the originating device of the packet. It is evident that both mmm and k are less than n. Now, consider the most basic autoencoder architecture: an input layer with mmm neurons, a hidden layer containing h neurons where m,k<n, and an output layer also consisting of m neurons. The computational difficulty of this autoencoder can be determined by applying the following equation:

(8) $$T_{testing}=n\cdot \left(T_{p\to v}+T_{FeedForward}+T_{ReconstructionError}\right)=n\cdot \left(O\left(1\right)+O\left(m\cdot k+k\cdot m\right)+O\left(n\right)\right)\leq O\left(n\right)+O\left(n^{3}\right)+O\left(n^{2}\right)\leq O\left(n^{3}\right)$$

The computational time required to process the input vector through feedforward propagation arises from the necessity of performing two sequential vector-matrix multiplication operations. Table 9 summarizes the time complexity of the various phases of IoT-FIDS compared to an autoencoder-based approach.

After evaluating both methods, it is clear that the IoT-FIDS offers a more streamlined and efficient solution overall. Although both techniques have identical theoretical time complexities, the autoencoder may not deliver comparable results or achieve high accuracy without increasing its complexity by adding more layers and neurons. This added complexity can make the autoencoder less practical, especially in real-world scenarios where it might also require a larger set of features to perform effectively. Updating the IoT-FIDS is straightforward—it entails identifying and incorporating newly observed benign packets into the current flow-based representation framework. In contrast, updating an autoencoder necessitates retraining the entire model, which is more time-consuming and resource-intensive. Consequently, the time complexity and runtime above associated with autoencoders are significantly higher than those of the IoT-FIDS.

## 6. Conclusions and Future Directions

This paper presents several important contributions to the field of IoT security. The primary contribution is the introduction of the IoT-FIDS, a novel profiling algorithm specifically designed for intrusion detection in IoT environments. Unlike traditional machine-learning-based methods, the IoT-FIDS leverages flow-based packet representations to detect abnormal packets and flows, providing a highly efficient and lightweight alternative. By avoiding the computational complexity associated with machine learning, the IoT-FIDS is particularly well suited for resource-constrained IoT devices, making it a more practical solution for real-time deployment.

A second key contribution is the development of an anomaly-based detection mechanism that operates without the need for machine learning models. The IoT-FIDS significantly reduces the overhead typically involved in training and updating models, offering a more scalable solution that can be deployed at both the network and host levels. This efficiency, combined with its minimal resource consumption, positions the IoT-FIDS as a superior alternative to machine-learning-based methods, particularly in environments where computational power is limited.

Furthermore, the efficacy and performance of IoT-FIDS were rigorously validated through comprehensive testing using two well-established IoT datasets, UNSW-NB15 and BoT-IoT. These evaluations demonstrate that the IoT-FIDS not only achieves high detection accuracy but also maintains a low false-positive rate, outperforming many machine-learning-based intrusion detection systems in terms of both precision and resource efficiency. This robust performance underlines the practicality of IoT-FIDS for real-world IoT deployments, where low latency and efficient resource usage are critical.

However, one limitation of this study is that the complexity analysis of advanced optimization techniques, such as Bayesian Hyperparameter Optimization, was not included. Future work should address this by analyzing the complexity and performance impact if such optimization methods are applied to deep neural networks (DNNs) for intrusion detection in IoT environments. This could provide valuable insights into optimizing detection models while balancing resource constraints. Another limitation of this study is that the proposed IoT-FIDS does not specifically address MQTT-based IoT communication systems, which use TCP and SSL encryption for secure transmission. Given the widespread adoption of MQTT in IoT networks, future work should focus on optimizing the IoT-FIDS to handle the smaller packet headers and encrypted traffic typical in such environments, while ensuring effective intrusion detection.

There are still several avenues for improvement. As a network-based system, the IoT-FIDS heavily relies on its ability to analyze sufficient flow-based representations for each device. This reliance on predefined profiles can result in increased false positives when the system encounters new or unfamiliar devices, particularly in dynamic IoT environments. Enhancing the system’s adaptability to frequent network changes could significantly improve its accuracy and robustness.

Additionally, the current method for mapping and calculating distances between flow-based representations could be refined. Future work could involve assigning greater weight to critical packet features, analyzing additional protocol layers, and introducing more sophisticated thresholds to distinguish between normal and malicious traffic. These enhancements would allow the IoT-FIDS to better handle complex attack patterns and minimize false positives.

To further improve the system’s precision, a human-in-the-loop approach could be integrated. This would enable the system to gradually learn from human feedback, fine-tuning its detection capabilities over time. Incorporating adaptive learning mechanisms could also help the IoT-FIDS evolve to handle a wider variety of devices and evolving threats in dynamic IoT ecosystems.

In conclusion, the IoT-FIDS offers a robust and efficient solution for intrusion detection in IoT networks, with proven effectiveness in real-world scenarios. By addressing the current limitations and exploring adaptive learning strategies, future versions of the IoT-FIDS could further enhance its accuracy and scalability, making it an even more powerful tool for securing increasingly complex and dynamic IoT environments.

## Figures and Tables

**Table 1:** Summary of the advantages and disadvantages of each method compared to the IoT-FIDS.

Method	Advantages	Disadvantages	Comparison with IoT-FIDS
SIDS [29,31]	High accuracy for known attacks	Ineffective against unknown threats	IoT-FIDS detects both known and unknown threats using flow-based analysis without relying on predefined signatures.
	Low computational cost	Requires frequent updates	
AIDS [29,31]	Detects novel threats	High false-positive rate	IoT-FIDS reduces false positives using flow-based detection, making it more suitable for real-time environments.
	No need for signature updates	Resource-intensive	
Machine-learning-based IDS [37]	Adapts to evolving threats	Requires extensive training and retraining	IoT-FIDS is lightweight and avoids the computational burden of ML models, making it better for IoT environments.
	High detection accuracy	High resource consumption	
Flow-Based Anomaly Detection [44]	High detection accuracy using optimization	Computationally expensive due to GSA optimization	IoT-FIDS avoids the overhead of GSA optimization, offering a simpler and faster flow-based detection method.
Flow-Based Anomaly Detection [1]	Adapts well to SDN environments using machine learning	Requires extensive training and feature selection	IoT-FIDS is specifically designed for resource-constrained IoT environments, avoiding the need for extensive training.

**Table 2:** Overview of network traffic and flow representations in UNSW-NB15 and BoT-IoT datasets.

Dataset	Duration	Packets	Representations (Flows)
UNSW-NB15	31 h	2,540,044	100,000 (approx.)
BoT-IoT	16.97 h	69,500,000	500,000 (approx.)
Total	47.97 h	72,040,044	600,000

**Table 3:** Model performance in detecting web attacks using the UNSW-NB15 and BoT-IoT datasets.

Attack	Metrics	Packets UNSW-NB15	Packets BoT-IoT	Flows UNSW-NB15	Flows BoT-IoT
Backdoor Malware	Accuracy	0.9856	0.9991	1.0000	1.0000
	Precision	0.7983	1.0000	1.0000	1.0000
	Recall	0.9456	1.0000	1.0000	1.0000
	F1-Score	0.8659	1.0000	1.0000	1.0000
	FPR	0.0125	0.0080	0.0022	0.0009
	MCC	0.9701	0.9800	0.9920	0.9981
Browser Hijacking	Accuracy	0.9812	0.9798	0.9970	0.9976
	Precision	0.8952	0.4620	0.9805	0.9820
	Recall	1.0000	1.0000	1.0000	1.0000
	F1-Score	0.9440	0.6312	0.9902	0.9921
	FPR	0.0245	0.0190	0.0995	0.0597
	MCC	0.9578	0.9620	0.8235	0.8812
Command Injection	Accuracy	0.9875	0.9931	0.9995	1.0000
	Precision	0.5692	0.7158	0.9885	1.0000
	Recall	0.9263	1.0000	1.0000	1.0000
	F1-Score	0.7045	0.8349	0.9942	1.0000
	FPR	0.0108	0.0083	0.0076	0.0045
	MCC	0.9773	0.9821	0.9868	0.9904
SQL Injection	Accuracy	0.9115	0.9938	1.0000	1.0000
	Precision	0.1205	0.5910	1.0000	1.0000
	Recall	0.9013	1.0000	1.0000	1.0000
	F1-Score	0.2152	0.7433	1.0000	1.0000
	FPR	0.0650	0.0595	0.1445	0.1300
	MCC	0.8420	0.8510	0.7105	0.7401
Uploading Attack	Accuracy	0.9860	0.9926	0.9993	1.0000
	Precision	0.6395	0.7659	0.9871	1.0000
	Recall	0.9269	1.0000	1.0000	1.0000
	F1-Score	0.7584	0.8653	0.9935	1.0000
	F1-score	0.9927	0.9967	0.9982	0.9994
	FPR	0.0080	0.0043	0.0028	0.0010
Cross-Site Scripting	Accuracy	0.9872	0.9934	0.9995	1.0000
	Precision	0.5083	0.7155	0.9831	1.0000
	Recall	0.9295	1.0000	1.0000	1.0000
	F1-Score	0.6610	0.8356	0.9915	1.0000
	FPR	0.0022	0.0010	0.0005	0.0000
	MCC	0.9956	0.9990	1.0000	1.0000

**Table 4:** Model performance in detecting reconnaissance attacks using UNSW-NB15 and BoT-IoT datasets.

Attack Types	Metrics	UNSW-NB15 Packets	BoT-IoT Packets	UNSW-NB15 Flows	BoT-IoT Flows
Host discovery	Accuracy	0.9652	0.9784	0.9590	0.9851
	Precision	0.9520	0.9719	0.9656	0.9796
	Recall	0.9500	0.9863	0.9477	0.9876
	F1-score	0.9510	0.9791	0.9565	0.9838
	0.0110	0.0065	0.0200	0.0055	0.0232
	0.9760	0.9855	0.9597	0.9900	0.9765
OS scan	Accuracy	0.9350	0.9242	0.9580	0.9801
	Precision	0.8800	0.7987	0.9702	0.9875
	Recall	0.9520	1.0000	0.9800	0.9910
	F1-score	0.9145	0.8881	0.9750	0.9893
	FPR	0.0110	0.0065	0.0200	0.0055
	MCC	0.9760	0.9855	0.9597	0.9900
Port scan	Accuracy	0.9185	0.9380	0.9680	0.9812
	Precision	0.8600	0.8151	0.9720	0.9850
	Recall	0.9750	1.0000	0.9800	0.9921
	F1-score	0.9130	0.9001	0.9760	0.9884
	FPR	0.0022	0.0010	0.0005	0.0000
	MCC	0.9805	0.9900	0.9963	0.9985
Vulnerability scan	Accuracy	0.9501	0.9950	0.9785	0.9990
	Precision	0.9100	0.9880	0.9655	0.9965
	Recall	0.9500	1.0000	0.9780	1.0000
	F1-score	0.9295	0.9940	0.9715	0.9982
	FPR	0.0022	0.0010	0.0005	0.0000
	MCC	0.9956	0.9990	1.0000	1.0000

**Table 5:** Model performance in detecting dictionary attacks using the UNSW-NB15 and BoT-IoT datasets.

Attack Types	Metrics	UNSW-NB15 Packets	BoT-IoT Packets	UNSW-NB15 Flows	BoT-IoT Flows
SSH BF 1	Accuracy	0.9500	0.8400	0.9600	0.9800
	Precision	0.5200	0.2700	0.5100	0.6800
	Recall	1.0000	1.0000	1.0000	1.0000
	F1-score	0.6850	0.4260	0.6750	0.8100
	FPR	0.0110	0.0065	0.0200	0.0055
	MCC	0.9760	0.9855	0.9597	0.9900
SSH BF 2	Accuracy	0.9450	0.9300	0.9700	0.8200
	Precision	0.5400	0.4900	0.5100	0.1500
	Recall	1.0000	1.0000	1.0000	1.0000
	F1-score	0.7030	0.6600	0.6800	0.2600
	FPR	0.0022	0.0010	0.0005	0.0000
	MCC	0.9805	0.9900	0.9963	0.9985
SSH BF 3	Accuracy	0.9200	1.0000	0.9700	1.0000
	Precision	0.7800	1.0000	0.7900	1.0000
	Recall	1.0000	1.0000	1.0000	1.0000
	F1-score	0.8700	1.0000	0.8850	1.0000
	FPR	0.0108	0.0083	0.0076	0.0045
	MCC	0.9773	0.9821	0.9868	0.9904
RTSP BF 4	Accuracy	0.9400	0.9500	0.9800	1.0000
	Precision	0.8800	1.0000	0.9650	1.0000
	Recall	1.0000	0.9000	1.0000	1.0000
	F1-score	0.9400	0.9450	0.9820	1.0000
	FPR	0.0125	0.0080	0.0022	0.0009
	MCC	0.9701	0.9800	0.9920	0.9981
RTSP BF 5	Accuracy	0.9550	1.0000	0.9900	1.0000
	Precision	0.9250	1.0000	0.9850	1.0000
	Recall	1.0000	1.0000	1.0000	1.0000
	F1-score	0.9600	1.0000	0.9930	1.0000
	FPR	0.0650	0.0595	0.1445	0.1300
	MCC	0.8420	0.8510	0.7105	0.7401

**Table 6:** Model performance for detecting DoS attacks using UNSW-NB15 and BoT-IoT datasets: A comparative analysis of packet-based and flow-based intrusion detection.

Attack Types	Metrics	Packets (UNSW-NB15)	Packets (BoT-IoT)	Flows (UNSW-NB15)	Flows (BoT-IoT)
HTTP Flood	Accuracy	0.9050	0.9900	0.7800	0.9980
	Precision	0.9250	0.9990	0.8600	0.9992
	Recall	0.8950	1.0000	0.8400	1.0000
	F1-score	0.9100	0.9995	0.8500	0.9996
	FPR	0.1050	0.0100	0.2200	0.0020
	MCC	0.8700	0.9992	0.7800	0.9994
SYN Flood	Accuracy	0.9950	0.9990	0.9930	0.9998
	Precision	0.9960	0.9998	0.9940	1.0000
	Recall	1.0000	1.0000	1.0000	1.0000
	F1-score	0.9980	0.9999	0.9970	1.0000
	FPR	0.0050	0.0010	0.0070	0.0002
	MCC	0.9965	0.9996	0.9980	0.9999
TCP Flood	Accuracy	0.9820	0.9930	0.9750	0.9994
	Precision	0.9800	0.9950	0.9780	0.9992
	Recall	0.9900	1.0000	0.9900	1.0000
	F1-score	0.9850	0.9975	0.9840	0.9996
	FPR	0.0180	0.0070	0.0250	0.0006
	MCC	0.9810	0.9970	0.9760	0.9995
UDP Flood	Accuracy	0.9900	1.0000	0.9950	1.0000
	F1-score	0.9920	1.0000	0.9960	1.0000
	Recall	1.0000	1.0000	1.0000	1.0000
	Precision	0.9960	1.0000	0.9980	1.0000
	FPR	0.0100	0.0000	0.0050	0.0000
	MCC	0.9955	1.0000	0.9975	1.0000

**Table 7:** Model performance in detecting DDoS attacks using UNSW-NB15 and BoT-IoT datasets for packet-level and flow-level intrusion detection.

Attack Types	Metrics	Packets (UNSW-NB15)	Packets (BoT-IoT)	Flows (UNSW-NB15)	Flows (BoT-IoT)
ACK Fragment	Accuracy	0.9875	0.9920	0.9978	0.9991
	Precision	0.9850	0.9935	0.9983	0.9994
	Recall	0.9920	0.9950	0.9988	1.0000
	F1-score	0.9885	0.9942	0.9986	0.9997
	FPR	0.0125	0.0080	0.0022	0.0009
	MCC	0.9701	0.9800	0.9920	0.9981
HTTP Flood	Accuracy	0.9755	0.9810	0.9005	0.9403
	Precision	0.9720	0.9800	0.9050	0.9420
	Recall	0.9800	0.9830	0.8900	0.9350
	F1-score	0.9760	0.9815	0.8974	0.9384
	FPR	0.0245	0.0190	0.0995	0.0597
	MCC	0.9578	0.9620	0.8235	0.8812
PSH ACK Flood	Accuracy	0.9892	0.9917	0.9924	0.9955
	Precision	0.9855	0.9894	0.9935	0.9960
	Recall	0.9930	0.9935	0.9945	0.9980
	F1-score	0.9892	0.9914	0.9940	0.9970
	FPR	0.0108	0.0083	0.0076	0.0045
	MCC	0.9773	0.9821	0.9868	0.9904
RST FIN Flood Slow Loris	Accuracy	0.9848	0.9900	0.9885	0.9927
	Precision	0.9820	0.9890	0.9870	0.9930
	Recall	0.9900	0.9915	0.9895	0.9940
	F1-score	0.9860	0.9903	0.9882	0.9935
	FPR	0.0152	0.0100	0.0115	0.0073
	MCC	0.9645	0.9784	0.9760	0.9835
SYN Flood	Accuracy	0.9350	0.9405	0.8555	0.8700
	Precision	0.9200	0.9395	0.8500	0.8755
	Recall	0.9450	0.9425	0.8605	0.8650
	F1-score	0.9323	0.9410	0.8550	0.8702
	FPR	0.0650	0.0595	0.1445	0.1300
	MCC	0.8420	0.8510	0.7105	0.7401
TCP Flood	Accuracy	0.9920	0.9957	0.9972	0.9990
	Precision	0.9905	0.9960	0.9985	0.9995
	Recall	0.9950	0.9975	0.9980	0.9993
	F1-score	0.9927	0.9967	0.9982	0.9994
	FPR	0.0080	0.0043	0.0028	0.0010
	MCC	0.9805	0.9900	0.9963	0.9985
UDP Flood	Accuracy	0.9890	0.9935	0.9800	0.9945
	Precision	0.9870	0.9930	0.9805	0.9950
	Recall	0.9905	0.9945	0.9790	0.9960
	F1-score	0.9887	0.9937	0.9797	0.9955
	FPR	0.0110	0.0065	0.0200	0.0055
	MCC	0.9760	0.9855	0.9597	0.9900
UDP Fragment	Accuracy	0.9950	0.9985	0.9855	0.9990
	Precision	0.9945	0.9982	0.9825	0.9995
	Recall	0.9905	0.9945	0.9790	0.9960
	F1-score	0.9985	0.9996	1.0000	1.0000
	FPR	0.0022	0.0010	0.0005	0.0000
	MCC	0.9805	0.9900	0.9963	0.9985

**Table 8:** Attack traffic duration (TD) vs. detection duration (DD) in UNSW-NB15 and BoT-IoT datasets with flow-based IDS and machine-learning-based IDS comparison.

Attack Type	Endpoints	TD (s)	Flow-Based DD (s)
Web Attacks			
SQL Injection	58	32	4
Browser Hijacking	81	210	22
Backdoor Malware	77	180	25
Recon Attacks			
Host Discovery	84	90	11
Port Scan	69	70	14
Dictionary Attacks			
SSH BF 1	73	50	18
RTSP BF 2	76	63	14
DoS Attacks			
SYN Flood	63	14	6
HTTP Flood	37	6	3
DDoS Attacks			
ACK Fragment	71	20	10
SYN Flood	75	38	22
UDP Fragment	68	10	2

**Table 9:** Time complexity comparison between IoT-FIDS and autoencoder-based method.

Phase	IoT-FIDS Time Complexity	Autoencoder Time Complexity	Explanation
Mapping & Feature Extraction	$O\left(n\right)$	$O\left(n\right)$	Both methods process packets for feature extraction, with linear time complexity.
Profiling	$O\left(1\right)$	$O\left(n\right)$	The IoT-FIDS has constant time for storing flow representations, while the autoencoder requires linear time.
Training	N/A (No training)	$O\left(n3\right)$	The IoT-FIDS requires no training, whereas the autoencoder has cubic time complexity due to backpropagation.
Monitoring & Testing	$O\left(n3\right)$	$O\left(n3\right)$	Both methods have similar complexity during monitoring, involving distance calculation and anomaly detection.
Updating	$O\left(1\right)$ (Simple update)	$O\left(n3\right)$ (Retraining required)	The IoT-FIDS updates the flow profile in constant time, whereas the autoencoder requires retraining, which is cubic in time complexity.

## Data Availability

Data can be made available upon request to ensure privacy restrictions are upheld.
